# A Systematic Critical Appraisal of the Methodological Quality of Systematic Reviews on the Effect of Autologous Platelet Concentrates in the Treatment of Periodontal Intraosseous Defects

**DOI:** 10.3390/ma13184180

**Published:** 2020-09-20

**Authors:** Agostino Guida, Gennaro Cecoro, Rosario Rullo, Luigi Laino, Massimo Del Fabbro, Marco Annunziata

**Affiliations:** 1 Maxillofacial and ENT Surgery Department, National Cancer Institute—IRCCS “Fondazione G. Pascale”, 80131 Naples, Italy; a.guida@istitutotumori.na.it; 2 Multidisciplinary Department of Medical-Surgical and Dental Specialties, University of Campania “Luigi Vanvitelli”, 80138 Naples, Italy; gennarocecoro@gmail.com (G.C.); rosario.rullo@unicampania.it (R.R.); luigi.laino@unicampania.it (L.L.); 3 Department of Biomedical, Surgical and Dental Sciences, University of Milan, 20122 Milan, Italy; massimo.delfabbro@unimi.it; 4 IRCCS Galeazzi Orthopedic Institute, 20161 Milan, Italy

**Keywords:** platelet concentrate, systematic review, meta-analysis, periodontal regeneration, intraosseous defect

## Abstract

The present study aimed to perform a systematic critical appraisal of the methodological quality of systematic reviews (SRs) on the effect of autologous platelet concentrates (APCs) in the treatment of periodontal intraosseous defects and to provide a synthesis of the main clinical findings available. An electronic and hand search was performed up to February 2020; 14 systematic reviews of randomized controlled trials (RCTs), of which 11 were meta-analyses, were included. Only one SR fully satisfied all 11 items of the AMSTAR (“A Measurement Tool to Assess Systematic Reviews”) checklist for methodological quality evaluation, 3 SRs were classified of high quality, 8 of medium quality, and 2 of low quality. There is some evidence on the beneficial additive effect of APCs in the surgical treatment of intraosseous defects when used alone or in combination with bone grafts. APCs did not show any advantage when used together with guided tissue regeneration (GTR) or enamel matrix derivative (EMD). Undertaking SRs which adhere to rigorous standards and protocols is strongly recommended. There are increasing data on the positive adjunctive effect of APCs in the surgical treatment of intraosseous defects but, due to the heterogeneity of the available primary studies, the quality of evidence remains rather low and further long-term well-designed RCTs are encouraged.

## 1. Introduction

Autologous platelet concentrates (APCs) have been applied since ’90 [1,2,3], as an autologous source of growth and differentiating factors to enhance the healing and regeneration of soft and hard tissues in different fields of oral surgery, including the treatment of periodontal intraosseous defects. APCs include different preparations, each with specific characteristics, that have been used in similar clinical applications. Platelet-rich plasma (PRP) and plasma rich in growth factors (PRGF) are considered as a “first generation” platelet concentrates. PRP was the first APC proposed in oral surgery [1,2], and is obtained by double centrifugation. Conversely, PRGF, first introduced by Anitua in 1999 [3], requires single centrifugation and, with respect to PRP, does not contain leukocytes and requires a smaller blood volume. Both PRP and PRGF need anticoagulants before centrifugation, and heterologous activators to trigger polymerization, which occurs rapidly, but also produces a rapid release of a large amount of growth factors, which tends to decrease considerably within a few days. Platelet-rich fibrin (PRF) is considered as a “second-generation” APC, obtained from 100% autologous sources. Its preparation requires just a single centrifugation stage in which polymerization occurs naturally, without the need for activators. In addition, PRF is characterized by a strong fibrous structure consisting of a three-dimensional fibrin scaffold and shows a sustained release of growth factors for several days [4,5,6]. Based on their leukocyte and fibrin content, APCs have been classified into four categories: P-PRP (pure platelet-rich plasma, no leukocytes, includes PRGF), L-PRP (leukocyte and platelet-rich plasma), P-PRF (pure platelet-rich fibrin, no leukocytes) and L-PRF (leukocyte and platelet-rich fibrin) [6].

The efficacy of platelet concentrates in periodontal regeneration is matter of debate in literature. In the last few years, several systematic reviews, with or without meta-analysis, examined the effect of APCs in the treatment of intraosseous periodontal defects, with heterogeneous findings.

Some authors considered exclusively PRP, exclusively PRF, or both. Some authors focused on the use of the APC alone, others included also bone grafts and barrier membranes or enamel matrix derivative (EMD). In some cases, different periodontal surgical procedures were included, on both soft and hard tissues, in others, the authors specifically focused on the intraosseous defects. Some authors reported beneficial effects of APCs in terms of clinical attachment level (CAL) gain and probing depth (PD) reduction, whereas others limited this effect only to the intraosseous defect fill.

The available reviews on the use of APCs in periodontal surgery were very heterogeneous and when the effects of treatment are not clear, it is difficult to provide clinical indications to practitioners.

Systematic reviews (SRs) of randomized controlled trials are considered the best source of evidence to support clinical decisions on interventions [7,8,9]. The way a systematic review is conducted can change the findings and recommendations. A critical assessment of available SRs may be essential to identify possible causes of heterogeneity and methodological problems of both reviews and primary studies, in order to guide future research to address specific research questions. The quality of SR can be critically and reproducibly assessed using specific grading instruments [10,11]. “A Measurement Tool to Assess Systematic Reviews” checklist (AMSTAR), for instance, was specifically developed for grading the quality of the reviews [12]. Only a few studies, to date, have been published in the periodontal field on the assessment of SRs’ quality using these established guidelines.

The aim of this meta-review, therefore, was to perform a systematic critical appraisal of the methodological quality of systematic reviews on the effect of autologous platelet concentrates in the treatment of periodontal intraosseous defects, and to provide a synthesis of the main clinical findings and recommendations deriving from the examined SRs.

## 2. Materials and Methods

### 2.1. Research Question

This critical appraisal of SRs was conducted and is reported, based on the Preferred Reporting Items for Systematic Reviews (PRISMA) statement (www.prismastatement.org) [13]. The concept of the study was registered in the PROSPERO International Prospective Register of Systematic Reviews (CRD42020178492). The research question addressed was the following: have the SRs, about the effect of APCs on the regeneration of periodontal intraosseous defects, been undertaken following a high methodological quality?

### 2.2. Literature Search

An extensive literature search up to March 2020 was conducted in the MEDLINE Database (via PubMed, and Books), Embase, and the Cochrane Database of Systematic Reviews (CDSR) using the search strategy depicted in Table 1. A hand search was also conducted on the major international journal of periodontics. Grey literature (including documents not controlled by commercial publishing organizations, such as internal reports, working papers, newsletters [14]) was also searched (https://www.greylit.org/; http://www.opengrey.eu/). The reference lists of all original research and review articles identified to be relevant to the subject were scanned for possible additional studies.

### 2.3. Inclusion and Exclusion Criteria

The inclusion criteria set were as follows:

- SRs and meta-analyses of either randomized controlled trials or controlled clinical trials on the effect of APCs on the treatment of periodontal intraosseous defects;
- SRs that evaluated the effect of any type of APC, either alone or in conjunction with other bio-materials or procedures, compared with a non-APC control.

The exclusion criteria were as follows:

- Narrative reviews;
- SRs including trials on non-intraosseous periodontal defects (or in which such data could not be extracted);
- SRs of in vitro or animal studies.

Only articles in English or Italian were included. No publication date restriction was applied.

### 2.4. Review Selection Process

Two reviewers (R.R. and L.L.) independently scanned the literature to identify the eligible articles, and in case of disagreements on the selection process, a consensus was reached through discussion. In the first stage, the titles and abstracts of potential papers researched were assessed, and papers that did not meet the inclusion criteria were discarded. In the second stage, the full text of selected papers was assessed and texts that did not meet the inclusion criteria were excluded with reason.

### 2.5. Data Extraction

Two independent reviewers (A.G. and G.C.) extracted data from the selected studies. Only data directly related to the regeneration of periodontal intraosseous defects were retrieved. Data were obtained exclusively from the meta-analyses (effect size) and not from the primary study reports. Disagreement was solved by discussion between the 2 authors to reach a consensus. For necessary missing data, the authors of the studies were contacted.

### 2.6. Methodological Assessment of the Systematic Reviews (SRs)

The methodological quality of the SRs included was assessed using the AMSTAR checklist. The methodological assessment was made by two reviewers (A.G. and G.C.) independently, after appropriate calibration. Any disagreement was solved by discussion. AMSTAR is a validated checklist comprising 11 items addressing important aspects of an SR. This tool addresses specific criteria when conducting systematic reviews, e.g., search strategy, inclusion and exclusion criteria, assessment of the methodological quality of trials included in the review. Each of the checklist items was scored with “2” (the assessed criterion was explicitly met in the SR), “1” (the criterion was not completely met), “0” (the criterion was not met), “CA” (cannot answer, the item is relevant but not described by the authors) or “NA” (not applicable, the item is not relevant, e.g., meta-analysis was not possible or was not attempted by the authors). The sum of all scores gives a total AMSTAR score. For each review, the total score could range from 0 (none of the criteria met) to 22 (all the 11 criteria met). For each item, the total score could range from 0 (none of the reviews satisfied the question) to 2x (number of included reviews). There is no guideline to classify the studies based on AMSTAR score so it has been suggested that a final score ranging from 15 to 22 corresponds to a high-quality SR; 8–14 corresponds to a medium-quality SR; 7 or less corresponds to a low-quality SR.

## 3. Results

### 3.1. Study Selection

**Figure 1:**
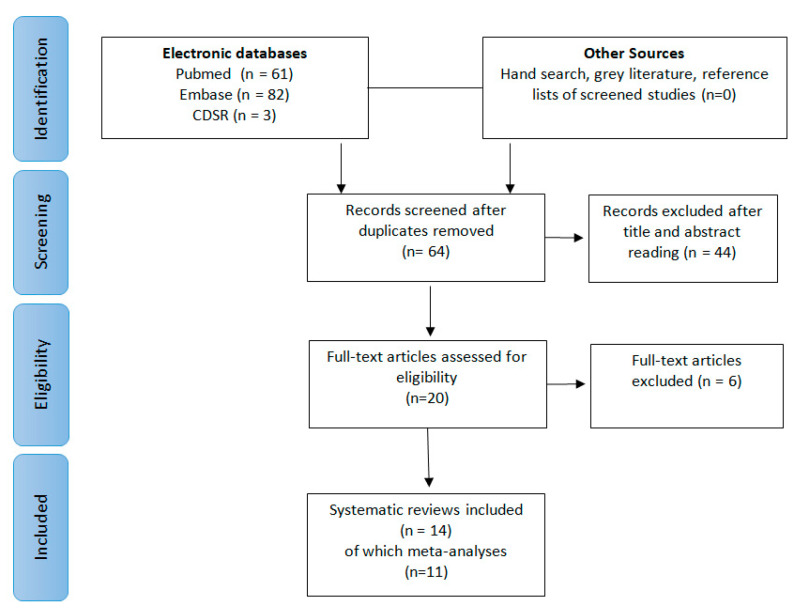
Preferred Reporting Items for Systematic Reviews (PRISMA) flow diagram of the study selection process.

The search in the electronic databases initially generated 64 potential papers. The other sources generated no additional paper. After duplicate removal and abstract assessment, 20 SRs were selected. Six articles were excluded after full-text reading because not systematically conducted [15], or because they pooled together different types of periodontal defects [16,17,18,19,20] (Table 2). Finally, 14 SR, 11 of which MA, were included for this critical assessment. The literature search process is depicted in Figure 1.

### 3.2. Characteristics of the SRs Included

Table 3 and Table 4 describes in detail the features of the SRs included. Four SR included only studies on PRP [21,22,23,24] (one of them included a study on “platelet pellet” that the authors considered as a PRP preparation) [21], five on PRF only [25,26,27,28,29] and five on platelet concentrates irrespective of their type (PRP/PRF and, in one case, PRP/PRF/PRGF) [22,30,31,32,33]. All the SRs included exclusively randomized controlled trials (RCT) on the use of APCs in the treatment of intraosseous defects. Four of them included also studies on other surgical applications of APCs (e.g., furcation defects, periodontal plastic surgery, alveolar socket preservation sinus elevation, etc.) [23,27,28,30], but in these SRs it was possible to clearly extract data about intraosseous defects. In all SRs the only difference between control and test groups was the presence of the APC. Three SRs included RCTs on APC alone as intervention group [25,27,29,33]. Six SRs included studies on both APCs used alone and added to other biomaterials [22,26,28,30,31,34]. Three SRs [21,23,24] included only studies in which PRP was added to biomaterials. In one SR [32] the studies included were exclusively on APCs (PRP and PRF) added to demineralized freeze-dried bone allograft (DFDBA). The follow-up of the studies included in the selected SRs ranged from 1 month in one study [26] to 5 years in another study [34], whereas most of the SRs included 6- or 9- to 12-month follow-up studies. All 11 MA were done on CAL gain, 8 on PD reduction [22,24,25,27,29,32,33,34], 5 on gingival marginal level change [25,29,32,33,34], 3 on intraosseous defect reduction [25,29,33], 4 on bone fill [22,27,29,32], 1 on bone reduction [32]. 4 MA performed sub-group analyses basing on the type of APC used (PRP/PRF) [32], the adjunct of a membrane (guided tissue regeneration (GTR)/no-GTR) [23,24,30], the experimental design (parallel groups/split-mouth design) [22,23,24,30], the follow-up period tested (3–6 months/9–12 months) [22]. Furthermore, one SR [22] performed separate meta-analyses based on the biomaterial or technique adjunctive to the APC (GTR/bone grafting (BG)/EMD/none), and another performed univariate meta-regression analyses of potential sources of heterogeneity (GTR, design, type of control) [24].

The overall effect size of the different outcomes is reported in Table 4. A direct comparison among the different SRs limited to CAL gain overall measurements is reported in Table 5.

### 3.3. Methodological Quality

The SRs were scored from 7 to 22 leading to a mean AMSTAR score of 12.6 ± 4.2 (standard deviation) corresponding to overall medium quality (Table 6). Only one SR fully satisfied all 11 items reaching an AMSTAR score of 22 [22]. Three SRs [21,31,34] were classified of high quality. All the others were of medium quality, excepting two [26,28] classified as low quality SRs. Only one review [22] completely met the first item (“a priori” design).

Most of the SRs (12/14) were conducted by two reviewers independently with consensus procedures for disagreements (item #2); 9 of 14 SRs used at least two electronic sources and one supplementary source (item #3), whereas only five explicitly search for “grey literature” (item #4).

Most of the SRs (8/14) clearly reported a list of the excluded studies (item #5) and all SRs reported the main characteristics of included studies (item #6), although only 5 completely met the criterion.

All SRs, excepting one [28], evaluated and documented the methodological quality of the included studies (item #7), although five SRs did not completely meet the criterion (score 1). In six SRs the results of the methodological rigor and scientific quality were considered in the analysis and the conclusions of the reviews and explicitly stated in formulating recommendations (item #8).

Six of the 10 MA used a test to ensure whether the studies were combinable and to assess their homogeneity (i.e., chi-squared test for homogeneity, or I^2^) and considered such aspects for methodological considerations (e.g., if heterogeneity exists, a random effects model should be used) (item #9). Similarly, in eight MA the publication bias was assessed by statistical test (e.g., Egger regression test) and/or graphical aids (e.g., funnel plot) (item #9), although two of them did not completely meet the criterion.

Finally, all the articles reported about potential sources of conflict of interest for the SR itself, but only two of them duly acknowledged the source of funding or support or the conflict of interest for each of the included studies.

### 3.4. Summary of Findings

Based on the results obtained from the systematic reviews reaching the highest quality scores, it can be concluded that there is some evidence for the beneficial additive effect of APCs in the surgical treatment of intraosseous defects when used alone or in combination with bone grafts, although the quality of evidence for such findings is low. By contrast, there is no evidence of any advantage when APCs are used together with GTR or EMD.

## 4. Discussion

With the widespread availability of scientific information, it may be difficult for clinicians to correctly interpret results and find evidence about clinical questions to guide clinical practice. This also applies to systematic reviews, the number of which has exponentially increased in recent years [35]. Systematic reviews of RCTs stay at the top of the evidence pyramid of scientific literature, but they need to be conducted following a very precise and well-defined methodology in order to be reliable and lead to consensus, recommendations, and clinical practice guidelines. For this reason, there is a need for a systematic critical appraisal of the methodological quality of systematic reviews on the basis of specific evaluation tools such as the AMSTAR [12].

In particular, the present AMSTAR-based assessment of SRs on the effect of APCs in the treatment of periodontal intraosseous defects revealed that some methodological aspects of the reviews could be improved. Out of 14 reviews assessed, in fact, only five [21,22,27,31,34] completely met (score 2) more than 50% of the AMSTAR criteria. Following this evaluation tool, several limitations of the SRs assessed were evidenced.

For example, almost all the SRs included did not fully meet an “a priori” design criterion. The execution of an SR adhering to acknowledged standards and guidelines, such as the PRISMA statement or the Methodological Expectations of Cochrane Intervention Reviews (MECIR) manual, is always strongly recommended. Only 5 of the 14 SRs included [22,24,27,29,33] referred to one of these guidelines. The use of a specialized framework, such as the PICO model [36] allows practitioners to formulate a well-focused research question, facilitating the literature search process to identify relevant evidence. PICO stands for Patient Problem (or Population), Intervention, Comparison (or Control), and Outcome, and its use is warmly recommended to correctly carry out an SR. Among the analyzed SRs, only 5 reported a PICO-based research question [22,27,28,31,34]. There are several public databases in which SRs can be registered, such as the International Prospective Register of Systematic Reviews of the National Institute for Health Research (PROSPERO), or the Cochrane Collaboration. Only two of the included SRs [22,33] referred to a registered protocol, and for one of them [33] the authors referred to ongoing submission. Systematic reviews should be registered at the protocol stage to enable comparison with already registered protocols, avoiding duplication, and to allow a post hoc comparison between the final publication and the planned protocol.

Most SRs performed a comprehensive literature search strategy with the use of supplementary sources of articles, such as a manual search in the main journals of periodontics, with analysis of the reference lists of the screened articles. However, extensive use of language limits, and limited use of grey literature sources, including the registers of clinical trials (e.g., http://www.clinicaltrials.gov), was found, which can imply a risk of publication bias [37,38,39].

Furthermore, only in five [21,22,24,28,32,34] of the included SRs a reproducible string of key-terms with Boolean operators was provided and only in two of them, specific strings for each database used were reported [22,27].

Finally, although most of the reviews make available a list of the studies excluded, it was limited only to the last phase of the review process, which is the full-text reading.

All these aspects, strongly limit the reproducibility of the review process in all its steps.

A complete report of the main characteristics of the included studies is of paramount importance to provide readers with an accurate report of the primary studies. All the analyzed SRs reported tables with such characteristics, however in more than half of SRs (those with score 1 of item #6 in Table 5) important details about participants (number, age, gender), operative protocol (preparation protocols of APCs) or results (mean values and standard deviation of baseline and follow-up values, and relative changes) were lacking.

Another aspect of central importance in carrying out an SR is the quality assessment of primary studies and its use to discuss results and make recommendations. It is usually performed using specific quality scoring tools or checklists, such as the Jadad scale [40], or the risk of bias tool of the Cochrane Collaboration [41]. All the analyzed SRs performed a methodological assessment of the primary trials. However, in four of them, the quality score was not reported for each study, and in one case [26], the authors used a minimum quality score as an “a priori” inclusion criterion, instead of performing such evaluation on all the included studies. Furthermore, only in one case [22] was the quality of evidence ranked based on specifically developed tools (i.e., Grading of Recommendations Assessment, Development and Evaluation, GRADE) [42]. Such aspects are of paramount importance to correctly interpret and weigh the validity of the results of each study [43], although the evaluation of each quality scoring tool is subjective. Despite calibration procedures and multiple assessment protocols, indeed, some discrepancies among different SRs can be found, which may influence the final interpretation of the results reported by the selected studies. Also when the scientific quality was correctly evaluated, we found that, in some cases, such evaluation was not explicitly used to analyze the results and to formulate scientific recommendations. In six of the analyzed SRs (score 1, item #8), for instance, the limits of the primary studies analyzed, and the relative recommendations referred to aspects not included in the quality assessment tool used. These elements regard statistical aspects (e.g., sample size calculation, appropriate statistical methods, analysis of confounding variables, etc.), or protocol aspects (e.g., appropriate follow-up duration, choice of the experimental groups, selection criteria, etc.). Probably, their evaluation could allow a more comprehensive “a priori” assessment of SRs’ scientific quality. Anyway, most of the SRs recommended performing better-designed RCT, with particular attention to methodological aspects such as randomization and allocation concealment.

Eleven of the 14 SRs undertook a MA of the main outcome measurements. MA is an element of paramount importance in an SR, allowing the treatment effect and its precision to be quantified. Nevertheless, if some methodological inaccuracy exists (e.g., heterogeneous trials pooled together), results and conclusions may be misleading. In some of the SRs analyzed, there were issues about the way the statistical aspects of the meta-analysis were described and presented. In particular, the evaluation of heterogeneity, as well as publication bias among studies, was not extensively reported and/or commented on [23,25,30,31,34]. In other cases, some discrepancy was found between figures and text [25], some data were missing, i.e., effect size [34] or significance values [25,32]. In one SR [34] the meta-analysis included only one study [44], with an inappropriate control group.

Interestingly, some SRs have performed sub-group [22,23,24,30,32] and meta-regression [24] analyses. However, the few primary studies available in some cases may considerably limit these attempts.

Finally, the reporting of potential sources of conflict of interest for each of the included studies remains one of the less met items in the quality assessment of the analyzed SRs.

Looking at the clinical findings from the SRs included, their conclusions are quite homogeneous. The evidence supporting the use of APCs in the treatment of periodontal intraosseous defects and its quality has been growing in the last few years but they are still limited.

As shown in Table 5, the standardized mean difference (SDM) of CAL gain for APCs alone (PRF, PRP, PRGF) ranged from 0.39 (95% confidence interval (CI), 0.35, 0.43, *p* < 0.00001) ([33] 16 studies), to 1.47 mm, 95% CI 1.11 to 1.82 mm; *p* < 0.00001 ([22], 12 studies). When added to a bone graft it was 0.72 mm, 95% CI 0.43 to 1.00 mm; *p* < 0.00001 ([22], 12 studies). A not significant overall effect, on the contrary, was reported for APCs added to GTR or to EMD [22,23,24,30,31]. Probably, as suggested by the authors, the reason can be found in the significantly high contribution of the membrane or EMD in the test group that may overwhelm and mask the positive influence of APC on the healing of the periodontal wound.

Overall, the authors agree on the beneficial effect of APC alone (mainly PRF) or in combination with different types of bone grafts (mainly PRP), but the clinical significance of the obtained results has been described as of limited value. This aspect was highlighted, in particular, by one SR [33], in which the authors followed a different approach for data analysis and interpretation, evaluating the “actual quantitative mean gains” (AQMG) for the main outcomes, intending to give more insight into clinical significance of conducted studies and a more direct evidential reference to the clinicians. Also calculating AQMG, the adjunctive effect of APCs in terms of clinical outcomes was considered negligible, because it was lower than a visible and perceptible gain value, which should be at least 2 mm [45].

Data are few and heterogeneous to speculate on the superiority of one type of APC compared to another (as well as on the superiority of one type of added graft compared to another). A direct comparison between different APCs was only rarely undertaken [46]. Furthermore, also indirect comparisons made by subgroups meta-analyses have not been attempted in the examined SRs, due to the low number and heterogeneity of available primary studies. Whereas, indeed, a consistent number of studies on PRF alone have been published in the last years, only one study on PRP alone was included in the examined SRs. Similarly, whereas most of studies on PRP included also an adjunctive graft, only very few studies on PRF + grafts are available to date. The reason for such a discrepancy might be the fluid nature of PRP, that limits its mechanical support when used alone, as opposed to the strong mechanical consistency of PRF, that, in turn, can limit its use in combination with grafts.

Although the similar clinical efficacy of PRF, PRP or PRGF in the treatment of intraosseous defects is shown in the few available comparative studies [46,47], some unique biological properties and advantageous procedural aspects of PRF compared to the other APCs have been suggested. PRF, indeed, shows gradual and prolonged release of growth factors, as well as antibacterial and anti-inflammatory effect due to the content of leucocytes [48,49,50,51,52]. Furthermore, PRF requires a single centrifugation, does not need any additive, and is characterized by an enhanced handling due to its more robust and long-lasting fibrin mesh [48,49,50].

Several other variables could affect the results of primary studies. For instance, different protocols exist for producing the same type of platelet concentrate, and they may affect the final composition and the biological properties. Lower centrifugation speed and time may result in higher leukocyte concentration, more even distribution of leukocytes throughout the PRF scaffold, and increased release of growth factors [4,51,53]. The clinical results, moreover, could be influenced by disease characteristics (grading of periodontitis), defect characteristics (number of walls, depth, width), surgical technique (modified Widman flap, Kirkland flap, papilla preservation flaps, single flap approaches).

RCTs analysing all these variables are still lacking and need to be specifically designed and carried out to achieve useful clinical indications. Also the follow-up period must be considered. It has been shown that the bone fill progressively goes on for a long time, reaching the highest level over 36 months [54]. For this reason, a more precise assessment of bone healing and regeneration would require longer follow-up studies.

Another systematic appraisal of SRs on the use of APCs for the treatment of periodontal intraosseous defects was published a few years ago [55], however some differences exist with the present overview. That study was not exclusively focused on intraosseous defects treatment but also on the furcations and gingival recessions treatment. It included 9 SRs on intraosseous defects (up to March 2016), whereas 14 SRs were identified in the present one (up to February 2020). Finally, some of the SRs included in that work were excluded in the present one after full-text reading [16,17,18,56], due to different selection criteria.

## 5. Conclusions

The methodological quality of the examined SRs was heterogeneous. The execution of SRs which adhere to acknowledged standards and guidelines, i.e., PRISMA or MECIR, and analyze the quality of evidence available in more depth employing specific instruments, e.g., GRADE, are strongly recommended

The results obtained from the systematic reviews reaching the highest quality scores suggest that the evidence on the positive adjunctive effect of APCs in the regeneration of intraosseous defects, alone (mainly PRF) or in combination with bone grafts (mainly PRP), has been increasing in recent years. Conversely, APCs did not show any advantage when used together with GTR or EMD. Due to paucity and heterogeneity of the available primary studies, it is not possible to speculate on the superiority of one type of APC or adjunctive graft compared to the others. The quality of evidence for such findings is still rather low and further long-term, multicentre well-designed RCTs are needed to validate these therapeutic approaches and provide evidence-based clinical recommendations.

## Figures and Tables

**Table 1:** Literature search strategy.

Database	Search String	No. of Items
PubMed	(((platelet OR plasma) AND (plasma OR derivative OR gel OR “growth factor” OR fibrin)) AND (periodontal OR periodontitis OR ((intrabony OR intraosseous) AND defect))) AND (“systematic review” OR “meta analysis”)	61
Embase	(‘platelet’/exp OR platelet OR ‘plasma’/exp OR plasma) AND (‘plasma’/exp OR plasma OR derivative OR ‘gel’/exp OR gel OR ‘growth factor’/exp OR ‘growth factor’ OR ‘fibrin’/exp OR fibrin) AND (periodontal OR ‘periodontitis’/exp OR periodontitis OR ((intrabony OR intraosseous) AND defect)) AND (‘systematic review’/exp OR ‘systematic review’ OR ‘meta analysis’/exp OR ‘meta analysis’)	82
CDSR	(((platelet OR plasma) AND (plasma OR derivative OR gel OR “growth factor” OR fibrin)) AND (periodontal OR periodontitis OR ((intrabony OR intraosseous) AND defect))	3

CDSR = Cochrane Database of Systematic Reviews.

**Table 2:** Excluded articles with reason of exclusion.

Study	Reason of Exclusion
Franchini et al., 2019 [20]	Pooled together data on intraosseous and non-intraosseous defects
Verma et al., 2017 [19]	Pooled together data on intraosseous and non-intraosseous defects
Roselló-Camps et al., 2015 [18]	Pooled together data on intraosseous and non-intraosseous defects and data on platelet poor plasma
Plachokova et al., 2008 [16]	Pooled together data on intraosseous and non-intraosseous defects
Martínez-Zapata et al., 2009 [17]	Pooled together data on intraosseous and non-intraosseous defects
Rock et al., 2013 [15]	Not a systematic review

**Table 3:** Characteristics of the included studies: search details.

Authors and Year	Focused Question/Aim	Search Strategy	Search Period	Key Words	Inclusion Criteria	Exclusion Criteria
Kotsovilis at al. 2010 [21]	What is the efficacy, with respect to clinical, radiographical and patient-centred outcomes, of combinations of platelet-rich plasma (PRP) with other therapeutic bioactive agents/procedures, compared with the efficacy of the same agents/procedures without the adjunctive use of PRP in the therapy of periodontal intraosseous defects in patients with chronic periodontitis and without systemic diseases that could potentially influence the outcome of periodontal therapy? PICO question: no	Electronic search: MEDLINE/PubMed, CENTRAL Manual search and other sources: specialized journals, references of relevant articles, proceedings, position papers, theses, contact with the authors to acquire missing, unclear or unpublished data. Language restrictions: none Publication date restrictions: yes Reference to an established guidelines: no Protocol registration: no	From 11/1997 to 09/2008	Key words provided: yes Repeatable search string: yes Specific search string for each database: no	Only randomized controlled trials (RCTs), either of a parallel group or of a split-mouth design; all patients included exhibit exclusively chronic periodontitis; all patients included, should have no systemic diseases; Presence of at least one experimental group, in which PRP was clinically applied as an adjunct to other therapeutic bioactive agents/procedures for the therapy of periodontal intraosseous defects; Presence of an appropriate non-PRP control group; Report of change in clinical attachment level between baseline and the end of follow-up period as the primary outcome variable and at least of change in probing pocket depth between baseline and the end of follow-up period as secondary outcome variable. Follow-up period of at least 6 months.	Mixed RCT design, including both parallel group and split-mouth design; use of historical control group; history of periodontal therapy within the preceding 12 month or less; periodontal intraosseous defect(s) extending into furcation area(s) or located around teeth presenting furcation involvement(s); patients receiving any medication reported to interfere with wound healing; patients with abnormal platelet counts; patients receiving antibiotics at the baseline of the RCT and/or during the previous 3 months or less; history of radiotherapy in the head and neck region of the patients; teeth presenting endodontic problems.
Del Fabbro et al., 2011 [23]	The aim of the present evidence-based systematic review is to determine whether the use of autologous platelet concentrates may affect the outcome of regenerative procedures for the treatment of periodontal defects and gingival recession. PICO question: no	Electronic search: MEDLINE/PubMed, CENTRAL. Manual search and other sources: specialized journals, references of relevant articles, contact with manufacturing companies for ongoing or unpublished studies. Clinical trials from public registers: no Grey literature: yes Language restrictions: none Publication date restrictions: none Reference to an established guidelines: no Protocol registration: no	Up to 09/2010	Key words provided: yes Repeatable search string: no Specific search string for each database: no	RCTs assessing the efficacy of platelet concentrates for healing and regeneration of hard and soft tissues in patients undergoing surgical procedures for the treatment of periodontal defects and gingival recession. Studies with a test group using platelet concentrates compared to a control group in which platelet concentrates were not used.	All other types of study designs, like case series, case reports, retrospective studies, technical studies, animal studies, and reviews; studies investigating the effect of platelet concentrates in surgical procedures involving implant therapy, like the maxillary sinus augmentation procedure or articles reporting on any other oral surgical intervention like tooth extraction, inlay and onlay grafts for the treatment of jawbone defects, treatment of odontogenic cysts, and periapical surgery.
Del Fabbro et al., 2013 [30]	The aim of this review was to systematically evaluate the effects of autogenous platelet concentrates as an adjunct to the surgical treatment of periodontal defects. PICO question: no	Electronic search: MEDLINE/PubMed, CENTRAL. Manual search and other sources: specialized journals, references of relevant articles, contact with manufacturing companies for ongoing or unpublished studies. Language restrictions: none Publication date restrictions: none Reference to an established guidelines: no Protocol registration: no	Up to 04/2012	Key words provided: yes Repeatable search string: no Specific search string for each database: no	RCTs and controlled clinical trials (CCTs) assessing the efficacy of platelet concentrates for healing and regeneration of hard and soft tissues in patients undergoing surgical procedures for the treatment of periodontal defects and gingival recession; no limitations regarding the number of patients treated; meta-analysis performed only if platelet concentrate was the only difference between test and control group.	All other types of study designs, like case series, case reports, retrospective studies, technical studies, animal studies, and reviews; Studies investigating the effect of platelet concentrates in surgical procedures involving implant therapy, like the maxillary sinus augmentation procedure or articles reporting on any other oral surgical intervention like tooth extraction, inlay and onlay grafts for the treatment of jawbone defects, treatment of odontogenic cysts, and periapical surgery.
Shah et al., 2014 [25]	The aim of the present evidence based systematic review and meta-analysis is to determine the clinical and radiographic outcomes of using platelet-rich fibrin (PRF) for the treatment of periodontal IBDs compared to open flap debridement (OFD). PICO question: no	Electronic search: MEDLINE/PubMed, EBSCO, CENTRAL. Manual search and other sources: none Language restrictions: English Publication date restrictions: yes Reference to established guidelines: no Protocol registration: no	From 01/2005 to 01/2013	Key words provided: yes Repeatable search string: yes Specific search string for each database: no	Studies (randomized and non-randomized clinical trial ndr) investigating the effect of PRF in the treatment of periodontal intraosseous defects; test group using PRF alone; control group with OFD alone; no limitations regarding the number of patients treated; follow-up of minimum 6 months.	Study designs such as case series, case reports, retrospective studies, technical studies, animal studies and reviews.
Hou et al., 2016 [24]	The aim of our study was to evaluate the efficacy of PRP in the surgical treatment of periodontal intrabony defects by comparing clinical outcomes between patients who received PRP as an adjunct to periodontal intrabony defect therapy and those who did not. PICO question: no	Electronic search: MEDLINE/PubMed, EMBASE, ISI Web of Science, CENTRAL. Manual search and other sources: references of relevant articles Language restrictions: none Publication date restrictions: none Reference to established guidelines: PRISMA Protocol registration: no	Up to 06/2015	Key words provided: yes Repeatable search string: yes Specific search string for each database: no	RCT in which an intervention group receiving PRP was compared with a control group not receiving PRP; patients included having no systemic illness or abnormal platelet counts that could affect the clinical outcome of periodontal therapy; follow-up period of at least 6 months.	Inadequate comparison of the results of PRP for the treatment of periodontal intrabony defects; PRP administered to both the intervention and control groups; use of a biologic material that would hamper meaningful comparisons; other article types, such as reviews, case reports, and animal studies.
Panda et al., 2016 [31]	What is the adjunctive effect of autologous platelet concentrates (APCs) over OFD in the treatment of periodontal intraosseous defects? PICO question: yes	Electronic search: MEDLINE/PubMed, EBSCO, CENTRAL. Manual search and other sources: specialized journals. Language restrictions: none Publication date restrictions: none Reference to established guidelines: no Protocol registration: no	Up to 06/2012	Key words provided: yes Repeatable search string: no Specific search string for each database: no	Clinical trials, either of a parallel group or of a splitmouth design; presence of experimental group in which APCs were clinically applied; presence of an appropriate non-APC control group; patients included in the RCT should present with intrabony defects (clinical attachment level (CAL) ≥4 mm and pocket depth (PD) ≥3 mm); patients included in the RCT should have no systemic diseases; report of clinical attachment level at baseline and at the end of the follow-up period as the primary outcome variable and PD or radiographic defect depth at baseline and at the end of follow-up period as the secondary outcome variable; articles having follow-up period of at least 9 months	RCT design, including both parallel group and splitmouth design; periodontal intrabony defects extending into furcation areas of teeth; intrabony defects extending apically with endodontic involvements.
Najeeb et al., 2017 [26]	In patients with intrabony periodontal defects, what is the effect of using PRF-based grafts on the clinical and radiographic outcomes? PICO question: no	Electronic search: MEDLINE/PubMed, Google Scholar, ISI Web of Science Manual search and other sources: references of relevant articles Language restrictions: English Publication date restrictions: yes Reference to established guidelines: no Protocol registration: no	From 1949 to 01/2016	Key words provided: yes Repeatable search string: no Specific search string for each database: no	Randomized control trials; restoration of bony periodontal defects; PRF as test intervention.	Letters to the editors, commentaries, animal studies, and in vitro studies
Castro et al., 2017 [27]	Does L-PRF promote periodontal wound healing in systemically healthy patients (ASA I) during periodontal surgery compared to traditional techniques? PICO question: yes	Electronic search: MEDLINE/PubMed, EMBASE, CENTRAL Manual search and other sources: references of relevant articles citation screening and expert recommendations Language restrictions: English Publication date restrictions: none Reference to established guidelines: PRISMA Protocol registration: no	Up to 07/2015	Key words provided: yes Repeatable search string: no Specific search string for each database: yes	Randomised controlled clinical trials (RCTs) or controlled clinical trials (CCTs); studies regarding periodontal surgery: intrabony defects, furcation defects and periodontal plastic surgery; L-PRF prepared following the protocol 2700 rpm/12 min or 3000 rpm/10 min; studies conducted in humans; no limitation in follow-up duration;	Case reports, case series, retrospective studies, studies regarding bone augmentation procedures, ridge preservation or implant surgery, other types of platelet concentrates (fibrin glues, PRP, PRGF, A-PRF, I-PRF…), animal studies, in vitro studies other applications of L-PRF in Medicine (Traumatology, Ophthalmology, Dermatology, etc.) or in Dentistry (Endodontics,…)
Miron et al., 2017 [28]	What indications has platelet rich fibrin been shown effective for tissue repair/regeneration of either soft or hard tissues in dentistry? PICO question: yes	Electronic search: MEDLINE/PubMed, EMBASE, ISI Web of Science, SciVerse, CENTRAL Manual search and other sources: relevant journals, references of relevant articles Language restrictions: English Publication date restrictions: none Reference to established guidelines: no Protocol registration: no	Up to 05/2016	Key words provided: yes Repeatable search string: yes Specific search string for each database: no	Human studies evaluating the comparative effects of PRF to an appropriate control or to another regenerative modality in human studies were included.	All human studies evaluating PRF in a case report or case series if controls were not present. All animal and in vitro studies.
Saleem et al., 2018 [34]	What are the vertical probing pocket depth reductions, the vertical clinical attachment level gains and the recession reduction at infra-bony defects at least 6 months after Regenerative Surgery with the adjunctive use of PRP as documented in RCTs, compared to the same clinical procedures and biomaterials performed without the use of PRP? PICO question: yes	Electronic search: MEDLINE/PubMed, EMBASE, LILACS, CENTRAL, Current Controlled Trials, ClinicalTrials.gov, World Health Organization International Trials Registry Platform, ISI Web of Knowledge (conference abstracts), Open Grey Manual search and other sources: relevant journals, references of relevant articles, contact with authors Language restrictions: English Publication date restrictions: none Reference to established guidelines: no Protocol registration: no	Up to 12/2016	Key words provided: yes Repeatable search string: yes Specific search string for each database: no	Study population: Studies were limited to human subjects older than 18 years and in good general health, with a diagnosis of chronic periodontitis and with at least one pair of specular infrabony defects. Type of interventions: guided tissue regeneration (GTR) surgical procedures with and without PRP will be the interventions considered for the comparative evaluation. Type of comparison: Infrabony defects […] by the same regenerative therapy without PRP that were considered the control group. Outcome measures: (i) probing pocket depth reduction (PPDRed mm), (ii) clinical attachment level gain (CAL Gain mm), (iii) recession reduction (RECRed). These were evaluated as the mean difference (mm) from the time of surgery until the end of the evaluation period not before 6 months. Types of Studies: randomized controlled clinical trials (RCTs) only;	Cohort studies or case-control studies. Case series and case reports. Studies considering individuals with a history of aggressive periodontitis or conducted on animal models.
Zhou et al., 2018 [32]	The aim of the present systematic review and meta-analysis was to evaluate and compare the clinical outcomes of enamel matrix derivative (EMD), PRP, PRF, and AM in conjunction with demineralized freeze-dried bone allograft (DFDBA) in patients with periodontal intrabony defects, which might have some guiding significance on clinical management strategy for the option of additional bioactive materials. PICO question: no	Electronic search: MEDLINE/PubMed, EMBASE, CENTRAL Manual search and other sources: references of relevant articles Language restrictions: none Publication date restrictions: none Reference to established guidelines: no Protocol registration: no	Up to 12/2017	Key words provided: yes Repeatable search string: yes Specific search string for each database: no	RCTs that compared the performances of DFDBA with or without one of the four bioactive materials (EMD, PRP, PRF, and AM) in patients with periodontal intrabony defects, with follow-up periods of ≥6 months.	Retrospective cohort studies, animal studies, in vitro studies, case reports, case series, and reviews.
Del Fabbro et al., 2018 [22]	To assess the effects of APCs used as an adjunct to periodontal surgical therapies OFD, OFD combined with BG, GTR, OFD combined with EMD) for the treatment of infrabony defects. PICO question: yes	Electronic search: MEDLINE/PubMed, EMBASE, LILACS, CENTRAL, ClinicalTrials.gov, World Health Organization International Trials Registry Platform, Grey Literature Report, Open Grey Manual search and other sources: relevant journals, references of relevant articles Language restrictions: none Publication date restrictions: none Reference to established guidelines: MECIR Protocol registration: Cochrane Database of Systematic Reviews, PROSPERO	Up to 02/2018	Key words provided: yes Repeatable search string: yes Specific search string for each database: yes	RCTs of both parallel and split-mouth design, involving patients with infrabony defects requiring surgical treatment. Studies had to compare treatment outcomes of a specific surgical technique combined with APC, with the same technique when used alone.	NR
Li et al., 2019 [29]	The aim of this updated meta-analysis was to systematically evaluate the additive effectiveness of autologous PRF in the treatment of intrabony defects of chronic periodontitis patients when used along with OFD in terms of clinical and radiological outcomes. PICO question: no	Electronic search: MEDLINE/PubMed, EMBASE, CENTRAL, ISI Web of Knowledge Manual search and other sources: references of relevant articles, contact with authors Language restrictions: English Publication date restrictions: none Reference to established guidelines: PRISMA Protocol registration: no	Up to 11/2017	Key words provided: yes Repeatable search string: no Specific search string for each database: yes	Trials had to be properly randomized; no additional agents or interventions confounded the comparison; contain patients with histologically proven intrabony defects of chronic periodontitis; patients included in the trials should have no systemic diseases that could potentially influence the outcome of periodontal therapy.	Studies only featuring comparisons of other types of chemotherapy regimens; early studies published as a series of articles from the same institution or author that contained significant overlapping data.
Baghele et al., 2019 [33]	The aim of the present meta-analysis is to evaluate actual quantitative mean gains of autologous platelet concentrates (PRF/PRP) in the treatment of intrabony defects in randomized controlled trials over and above that of OFD. The focused question for our meta-analysis is, “Whether there is any clinically significant advantage of using autologous platelet concentrates (PRF/PRP) along with OFD in intrabony defects, as represented by various clinical and radiographic periodontal parameters when compared to use of OFD alone?” PICO question: no	Electronic search: MEDLINE/PubMed, EMBASE, EBSCO, Google Scholar Manual search and other sources: relevant journals, references of relevant articles Language restrictions: English Publication date restrictions: none Reference to established guidelines: PRISMA Protocol registration: submission to PROSPERO (not verified)	Up to 05/2017	Key words provided: yes Repeatable search string: no Specific search string for each database: no	Human randomized clinical trials, either of a parallel group or of a split-mouth design, reporting adequate and readable data from ≥10 subjects/osseous defects in the PRP or PRF group. A randomized controlled clinical trial where one of the groups received autologous PRF/PRP. The comparator group can be of any treatment modality but only OFD alone was considered for analyses. The patients included in the RCT had no systemic illness or abnormal platelet counts that could affect the clinical outcome of periodontal therapy Periodontal intrabony defects with radiographic IBD ≥3 mm with corresponding CAL ≥5 mm were included. All the defects irrespective of mentioned number of walls (1, 2 or 3 walled defects) were included. Studies determining at least one of these variables were included: the clinical attachment levels (CALs), the depth of intrabony defect, and the probing pocket depths (PPDs) at baseline and final follow-up of at least 6 months	Studies mentioning furcation invasions of teeth. Study designs such as case series, case reports, retrospective studies, in vitro studies, animal studies, reviews, and meta-analyses.

PICO = Patient Problem (or Population), Intervention, Comparison (or Control), and Outcome; IBDS = intrabony defects; ASAI = score I of the American Society of Anesthesiologists classification of Physical Health; AM = amnion membrane; MECIR = Methodological Expectations of Cochrane Intervention Reviews.

**Table 4:** Characteristics of the included studies: main findings.

Authors and Year	Studies Included (Only Studies on Intrabony Defects Considered)	APCs Evaluated	Groups	Follow-Up Range (Months)	Meta-Analysis	Total Defects Test/Control	Overall SMD (95% CI)	Conclusions
Kotsovilis et al., 2010 [21]	10 RCTs	PRP (including PP)	PRP + graft PRP + ABBM vs. ABBM PRP + ABBM + EMD vs. ABBM + EMD PPR + BG vs. BG PRP + β-TCP vs. β-TCP PRP + DFDBA vs. DFDBA PRP + HA vs. HA PRP + GTR PRP + BM + ePTFE-GTR vs. BM + ePTFE-GTR PRP + BM + COL-GTR vs. BM + COL-GTR PRP+ β-TCP + ePTFE-GTR vs. β-TCP + ePTFE-GTR Other comparisons PP + PAM-GTR vs. BG + PAM-GTR	6–12	No	184/183	NA	General conclusions Most RCTs selected generally demonstrate appropriate methodology with regard to the majority of quality criteria. However, most of studies selected are lacking sample size calculation, and in certain RCTs randomization and allocation concealment methods are not clearly adequate. The selected RCTs differ in their design with regard to therapeutic bioactive agents/procedures combined with PRP for the therapy of periodontal intraosseous defects. The amount of data currently available for each combination of PRP with other therapeutic bioactive agents/procedures could be regarded as limited. Publication bias and its specific types, language bias and time-lag bias, might possibly lead to an overestimation of the efficacy of the adjunctive use of PRP. Specific conclusions The clinical use of PRP is an entirely safe procedure, causing no adverse events or postoperative complications. Diverse outcomes (positive and negative) have been reported for the efficacy of PRP combined with various therapeutic bioactive agents/procedures, reflecting the limited and heterogeneous data available and possibly suggesting that the specific selection of agents/procedures combined with PRP could be important. Implications for research and clinical practice Randomized controlled clinical trials should include an appropriate (concurrent with the experimental group) non-PRP control group and longer follow-up periods. Consensus on an appropriate methodology for PRP preparation seems to be required. A specific protocol for the clinical use of PRP cannot be recommended at present.
Del Fabbro et al., 2011 [23]	10 RCTs	PRP	PRP + graft PRP + ABBM vs. ABBM (2) PRP + HA vs. HA PRP + BG vs. BG PRP +DFDBA vs. DFDBA PRP + ABBM + EMD vs. ABBM + EMD PRP + GTR PRP + ABBM + GTR vs. ABBM + GTR (2) PRP + β-TCP + GTR vs. β-TCP + GTR (2) Other comparisons (not meta-analysed): PRP+ABBM+GTR vs. GTR PRP + ABBM + GTR vs. none PRP + β-TCP + GTR vs. β-TCP PRP+GTR vs. GTR + BG and PRP + β-TCP vs. β-TCP PRP + ABBM + GTR vs. ABBM + GTR PRP + β-TCP vs. β-TCP	6–12	Yes	307/295	PRP + graft or GTR vs. graft or GTR CAL Gain (10): 0.50 mm (95% CI: 0.12–0.88 mm, *p* = 0.01) Sub-groups: GTR (4): 0.04 mm (95% CI: −0.33, 0.41 mm, *p* = 0.75) no-GTR (6): 0.84 mm (95% CI: 0.27, 1.42 mm, *p* = 0.004) P (7):0.39 mm (95% CI: −0.01, 0.79 mm) SM (3): 0.80 mm (95% CI: 0.10, 1.50 mm)	Platelet-rich plasma may be advantageously used as an adjunct to grafting procedures, but not in combination with GTR, for the treatment of intrabony defects
Del Fabbro et al., 2013 [30]	18 RCTs	PRP PRF	APC alone PRF vs. OFD (3) PRP vs. OFD PRP + graft PRP + graft PRP + ABBM vs. ABBM (3) PRP + HA vs. HA PRP + BG vs. BG PRP + DFDBA vs. DFDBA PRP + ABBM + EMD vs. ABBM + EMD PRP + HA + β-TCP vs. HA + β-TCP PRP + β-TCP vs. β-TCP (2) PRP + GTR PRP + β-TCP + GTR vs. β-TCP + GTR (2) PRP + ABBM + GTR vs. ABBM + GTR (2) PRP + MTB + GTR vs. MTB + GTR Other comparisons (not meta-analysed): PRF + HA vs. OFD PRP + ABBM + GTR vs. GTR PRP + ABBM + GTR vs. none PRP + GTR vs. GTR + BG PRP + β-TCP + GTR vs. β-TCP PRP + β-TCP vs. β-TCP PRP + ABB + GTR vs. ABB + GTR PRP + β-TCP vs. β-TCP	6–12	Yes	327/305	PRP + graft or GTR vs. graft or GTR CAL gain (18): 0.67 mm (95%: 0.55, 0.78 mm), *p* < 0.001 Subgroups: P (11): 4.70% (95% CI: 0.97, 8.43%, *p* = 0.01) SM (7): 12.22% (95% CI:7.54, 16.90%, *p* < 0.001) GTR (5): 2.77% (95% CI: −2.23, 7.77%, *p* = 0.28) non-GTR (13): 9.17% (95% CI:5.39, 12.94%, *p* < 0.001) APC vs. OFD CAL gain (4): 4.96% (95% CI: −1.65, 11.58%, *p* = 0.14)	The use of APCs may exert a positive adjunctive effect for the treatment of intrabony defects when used in combination with graft materials, but not with GTR. No significant adjunctive benefit of APCs could be demonstrated for the treatment of gingival recession and furcation defects. A standardization of study design and clinical protocols is needed in future studies in order to gain more insight into the true effect of APCs in periodontal regeneration.
Shah et al., 2014 [25]	5 RCTs	PRF	PRF alone PRF vs. OFD (5) Other comparisons not meta-analysed: PRP vs. OFD PRF vs. PRP PRF vs. PRF + HA PRF + HA vs. OFD	9–12	Yes	103/102	PRF vs. OFD CAL gain (5): 0.95 (95%: IC: 0.20–1.71, *p* < 0.001 *) PDred (5): 1.10 (95% CI: 0.56–1.64, *p* < 0.001 *) IBDred (5): 2.33 (95% CI: 1.43–3.23, *p* < 0.001 *) RECinc (5): −0.47 (−1.52–0.58, *p* > 0.05) *	This meta-analysis showed clinically significant improvements in the periodontal parameters like CAL gain, IBDred, PDred when intrabony defects were treated with PRF alone compared to OFD. As PRF is easy to obtain autologous material, effective and user friendly, can be more widely used in periodontal regeneration.
Hou et al., 2016 [24]	12 RCTs	PRP	PRP + graft PRP + HA vs. HA PRP + ABBM vs. ABBM (3) PRP + BG vs. BG PRP + DFDBA DFDBA (2) PRP + β-TCP + HA vs. β-TCP + HA PRP + GTR PRP + β-TCP + GTR vs. β-TCP + GTR (2) PRP + ABBM + GTR vs. ABBM + GTR (2) Other comparisons (not meta-analyzed): PRP + HA vs. HA PRP + β-TCP vs. β-TCP (2)	6–13	Yes	217/215	PRP + graft or GTR vs. graft or GTR CAL gain (12): 0.76 mm (95% CI: 0.34, 1.18 mm, *p* = 0.0004) PDred (12): 0.53 mm (95% CI: 0.21, 0.85 mm, *p* = 0.001) Subgroups: GTR (4): 0.08 mm (95% CI: −0.30, 0.46 mm, *p* = 0.67) no-GTR (8): 1.22 mm, 95% CI: 0.88, 1.57 mm, *p*<0.00001) P (7): 0.45 mm (95% CI: −0.05, 0.94 mm, *p* = 0.08) SM(5): 1.20 mm (95% CI: 0.72, 1.69 mm, *p* < 0.00001)	The adjunctive use of PRP together with conventional grafting procedures may be a beneficial treatment approach. However, when combined with the use of a regenerative technique, such as GTR, the beneficial effect of PRP on the treatment of intrabony defects is negligible.
Panda et al., 2016 [31]	15 RCTs	PC (PRP and PRF)	APC alone PRF vs. OFD (4) PRP vs. OFD (1) PRP + graft PRP + TMB vs. TMB PRP + β-TCP vs. β-TCP PRP + ABBM vs. ABBM (2) PRP +DFDBA vs. DFDBA PRP + BG vs. BG PRP + HA vs. HA PRP + GTR PRP + β-TCP + GTR vs. β-TCP + GTR (2) PRP + ABBM + GTR vs. ABBM + GTR (2) Other comparisons (not meta-analyzed): PRF + HA + OFD vs. OFD	9–12	Yes	NA	PRFvsOFD (4) CAL gain: 1.48 (95% CI: 1.16, 1.79), *p* = 0.003 * PRP + graft vs. graft (7) CAL gain: 2.00 (95% CI: 1.68, 2.32), *p* < 0.001 * PRP + GTR vs. GTR (4) CAL gain: 0.03 (95% CI: −0.32, 0.37), *p* = 0.74 * PRP vs. OFD (1) CAL gain: 0.10 (95% CI: −0.09; 0.29), *p* = 0.30 *	Based on the results obtained from the present systematic review it can be concluded that the evidence on the beneficial additive effect of APCs in surgical treatment of intrabony defects has been increasing in recent years. Platelet concentrates may be advantageously used as a cost-effective adjunct to surgical regenerative therapy, even in combination with bone grafts, although they did not show any advantage when used together with GTR. Moreover, platelet-rich fibrin proved to be effective as a sole regenerative material for treatment of intrabony defects, in combination with OFD. Further long-term, multicentre clinical trials are to be carried out to validate these treatment strategies in evidence-based clinical practice.
Najeeb et al., 2017 [26]	13 RCTs	PRF	APC alone PRF vs. OFD (6) PRP vs. OFD APC + graft PRF + DFDBA vs. DFDBA (2) PRF + HA vs. HA PRF + ABBM vs. ABBM PRGF + ABBM vs. ABBM Other comparisons: PRF + ABBM vs. PRF PRF + DFDBA vs. PRF PRF vs. EMD PRF + MF vs. OFD MF vs. OFD ABG vs. OFD	1–12	No	NA	NA	The PRF when combined with OFD, produces better outcomes compared to the OFD alone. The regenerative potential of PRF results in better augmentation and regeneration of periodontal bone defects. In addition, PRF may augment the regenerative potential of bone grafts. However, more long-term and well-designed clinical trials are needed to ascertain the clinical efficacy of PRF and PRF containing bone grafts.
Castro et al., 2017 [27]	13 RCTs	L-PRF	PRF alone PRF vs. OFD (6) Other comparisons (not meta-analyzed): PRP vs. OFD PRF vs. ABBM PRF vs. DFDBA (3) PRF vs. EMD PRF vs. HA Other comparisons: PRF vs. ABG	6–12	Yes	129/129	PRF vs. OFD CAL gain (6): 1.2 mm (95% CI: 0.5, 1.9, *p* < 0.001) PDred (6): 1.1 mm (95% CI: 0.6, 1.6, *p* < 0.001) BF (6): 1.7 mm (95% CI: 1.0, 2.3, *p* < 0.001)	Favourable effects on hard and soft tissue healing and postoperative discomfort reduction were often reported when PRF was used. Nevertheless, standardization of the protocol is needed to obtain an optimal effect of PRF in regenerative procedures. Correct handling of PRF as well as the use of enough clots/membranes per surgical site might be crucial to obtain benefits from this technique. This biomaterial can be taken into consideration due to its reported good biological effects, low costs and ease of preparation.
Miron et al., 2017 [28]	10 RCTs	PRF	PRF alone PRF vs. OFD (6) PRF + graft PRF + HA vs. HA PRF + DFDBA vs. DFDBA PRF + MF vs. MF PRF + GTR PRF + GTR vs. GTR Other comparison: PRF vs. DFDBA	6–12	No	NA	NA	This systematic review demonstrates the widespread use of PRF in dentistry in various clinical settings. Although this regenerative modality remains unfamiliar to many clinicians, the evidence supporting its use has accumulated over the years, demonstrating its ability to improve tissue regeneration. The combination of PRF with regenerative therapy has been shown to be most promising for periodontal repair of intrabony […] defects […]. […] Nevertheless, its ease of use, combined with its low cost and autologous source, makes it an ideal biomaterial worth further investigation across a variety of surgical procedures in dentistry.
Saleem et al., 2018 [34]	15 RCTs	PRP	PRP + graft PRP + ABG vs. ABG PRP + HA vs. HA PRP + HA + β-TCP vs. HA + β-TCP PRP + ABBM vs. ABBM (2) PRP + DFDBA vs. DFDBA PRP + GTR PRP + ABBM + GTR vs. ABBM + GTR (2) PRP + β-TCP + GTR vs. β-TCP + GTR PRP + ABBM + GTR vs. GTR PRP + EMD PRP + EMD + ABBM vs. EMD + ABBM (2) Other comparisons: PRP + ABBM + GTR vs. OFD Other comparisons (not meta-analyzed): PRP vs. OFD PRP + EMD + ABBM vs. EMD + ABBM PRP + β-TCP vs. β-TCP PRP + TMB vs. TMB	6–60	Yes	NA	NA	The adjunctive use of PRP in the regenerative treatment of infrabony defects can be considered as an affordable technique to get a better CAL gain and PDred in the surgical treatment of periodontal infrabony defects. Anyway, the limitations of the provided studies are the lack of baseline data regarding the defect size and their morphology, the absence of reports of other relevant clinical outcomes, as the bone fill, and the heterogeneity between studies. On the basis of this systematic review, the regeneration/ repair of infrabony defects would favour the use of adding PRP to a simple surgical repositioned flap technique, like in the OFD, with the use of bone grafts (xenografts, HA, or TCP). No better results would be achievable using combinations with biomodulators (Emdogain) or membranes, the PRP just would act as a biomodulator itself. In a biological sense, this observation would state for the biomolecular signalling action between PRP and the surrounding cellular environment that any membrane could interrupt or modify. The use of bone grafts would state as a blood clot stabilizer enhancing the osteoinductive properties of the PRP itself. Future Research/Observations According to the main reported pitfalls, future studies should be aimed first, designed according to RCT schemes in order to provide clinical evidences. A comparison between a surgical flap approach alone and the adjunctive use of PRP would be needful in order to explore the role of growth factors alone in periodontal regeneration and the healing process, as well as the radiographic bone level assessment before and after treatment, as they represent a critical parameter in success assessment. In order to explore which growth factor would be better suited in periodontal procedures, a multiple-arm RCT would be needful comparing PRP with other blood-derived agents available as well as with the different techniques adopted to deliver it.
Zhou et al., 2018 [32]	9 RCTs	PRP, PRF	APC + graft PRP + DFDBA vs. DFDBA (4) PRF + DFDBA vs. DFDBA (2) Other comparisons: EMD + DFDBA vs. DFDBA AM + DFDBA vs. DFDBA	6–12	Yes	PRP+DFDBA vs. DFDBA (76/76) PRF+DFDBA vs. DFDBA (40/40)	Subgroups: PRP+DFDBA vs. DFDBA PDred (4): 0.47 95% CI: 0.14, 0.80, SS (*p*-value NA) CAL gain (4): 0.80 95% CI: 0.27, 1.32, SS (*p*-value NA) RECred (4): 0.45 95% CI: −0.18, 1.09, NSS (*p*-value NA) BF (4): 0.71 95% CI: 0.13, 1.29, SS (*p*-value NA) BR (3): −0.13 95% CI: −0.48, 0.21, NSS (*p*-value NA) PRF+DFDBA vs. DFDBA PDred (2): 0.88 95% CI: 0.41, 1.34, SS (*p*-value NA) CAL gain (2): 1.61 95% CI: 1.10, 2.12, SS (*p*-value NA) RECred (2): 0.77 95% CI: 0.31, 1.22, SS (*p*-value NA) BF: (2): 0.89 95% CI: −0.46, 2.24, NSS (*p*-value NA) BR (2): −0.18 95% CI: −0.62, 0.26, NS (*p*-value NA)	Within the limitation of this analysis, it is indicated that PRF exerts the most significant adjunctive effect on soft tissue healing, while PRP exhibits a unique impact on hard tissue reconstruction in the treatment of periodontal intrabony defect. […] Therefore, it seems reasonable to suggest that the autologous PRF/PRP could be taken as a preferred adjunct to promote periodontal regeneration due to its proven good biological effects, low costs, and ease of preparation. Nevertheless, standardization of the protocol for the preparation and application of PRF/PRP is needed to obtain an optimal effect in regenerative procedures.
Del Fabbro et al., 2018 [22]	38 RCTs	PRP PRGF PRF	APC alone APC vs. OFD (12) APC + graft APC + graft vs. graft (17) APC + GTR APC+GTR vs. GTR (7) APC + EMD APC + EMD vs. EMD (2)	3–12	Yes	APC + OFD vs. OFD (255/255) APC + OFD + graft vs. OFD + graft (284/284) APC + GTR vs. GTR (124/124) APC + EMD vs. EMD (38/37)	APC vs. OFD (F-U 9–12 m): Pdred(12): 1.29 mm (95% CI: 1.00 1.58 mm; *p* < 0.00001 P(7): 0.99, 95%CI0.90 to 1.07; *p* < 0.00001 SM(5): 1.86, 95% CI 1.07 to 2.66; *p* < 0.00001 CAL gain (12): 1.47 mm, 95% CI 1.11 to 1.82 mm; *p* < 0.00001 P(7): 0.99, 95%CI0.84 to 1.14; *p* < 0.00001 SM (5): 2.36,95% CI 1.19 to 3.54; *p* = 0.00008 BF (9): 34.26%, 95% CI 30.07% to 38.46%; *p* < 0.00001 P (7): 35.77%, 95% CI 31.20% to 40.35%; *p* < 0.00001 SM (2): 27.32%, 95% CI 20.92% to 33.72%; *p* < 0.00001 *APC + graft vs. graft* APC + graft vs. graft (all F-U): PDred (17): 0.54 mm,95% CI 0.33 to 0.75 mm; *p* < 0.00001; P (5): 0.81, 95% CI 0.58 to 1.03; *p* < 0.00001 SM (12): 0.47, 95% CI 0.24 to 0.71; *p* = 0.000099 CAL gain (17): 0.72 mm, 95% CI 0.43 to 1.00 mm; *p* < 0.00001 P (5): 0.89, 95% CI 0.49 to 1.29; *p* = 0.000012 SM (12): 0.67, 95% CI 0.35 to 0.99; *p* = 0.000047 BF (11): 8.10% 95% CI 5.26 to 10.97; *p* < 0.00001 P (3): 9.66%,95% CI 5.39% to 13.94%; *p* < 0.00001 SM (8): 7.73%, 95% CI 4.50% to 10.97%; *p* < 0.00001 *APC + graft vs. graft* (F-U 3–6 m): PDred (11): 0.62, 95% CI 0.30 to 0.94; *p* = 0.00015 P (1): 0.84, 95% CI 0.60 to 1.07; *p* < 0.00001 SM (10): 0.58, 95% CI 0.25 to 0.92; *p* = 0.00067; CAL gain (11): 0.47, 95% CI 0.11 to 0.84; *p* = 0.012 P (1): 1.00, 95% CI 0.93 to 1.07; *p* < 0.00001 SM (10): 0.40, 95% CI 0.02 to 0.77; *p* = 0.039; BF (6): 4.76%, 95% CI 1.27% to 8.25%; *p* = 0.0076 P (1): 10.00%, 95% CI4.90% to15.10%; *p* = 0.00012 SM (5): 3.59%, 95% CI 0.13% to 7.05%; *p* = 0.042 *APC + graft vs. graft (F-U 9–12 m)*: PDred (10): 0.50, 95% CI 0.31 to 0.69; *p* < 0.00001 P (4): 0.58, 95% CI 0.09 to 1.06; *p* = 0.020 SM (6): 0.49, 95% CI 0.26 to 0.72; *p* = 0.000039 CAL gain (6): SM 0.84, 95% CI 0.62 to 1.06; *p* < 0.00001 BF (6): 9.99%, 95% CI 6.44% to 13.55%; *p* < 0.00001 P (2): 8.87%, 95% CI 1.03% to 16.71%; *p* = 0.027 SM (4): 10.16%, 95% CI 6.18% to 14.14%; *p* < 0.00001 APC + GTR vs. GTR *APC + GTR vs. GTR (all F-U)*: PDred (7): 0.92, 95% CI −0.02 to 1.86; *p* = 0.054 P (3): 0.25, 95% CI −0.15 to 0.64; *p* = 0.22 SM (4): 1.52, 95% CI 0.54 to 2.51; *p* = 0.0024 CAL gain (7): 0.42, 95% CI −0.02 to 0.86; *p* = 0.060 P (3): 0.09, 95% CI −0.32 to 0.50; *p* = 0.66 SM (4): 0.67, 95% CI 0.20 to 1.14; *p* = 0.0048 *APC + GTR vs. GTR (F-U 3–6 m)*: PDred (3): SM: 1.07 (95% CI −0.71 to 2.86) *p* = 0.24 CAL gain (3): SM: 0.54, 95% CI 0.18 to 0.89; *p* = 0.0031 *APC + GTR vs. GTR (F-U 9–12 m)*: PDred (5): 0.68, 95% CI −0.66 to 2.02; *p* = 0.32 P (3): 0.25, 95% CI −0.15 to 0.64; *p* = 0.22 SM (2): 1.53, 95% CI −0.85 to 3.91; *p* = 0.21 CAL gain (5): 0.27, 95% CI −0.39 to 0.93; *p* = 0.42 P (3): 0.09, 95% CI −0.32 to 0.50; *p* = 0.66 SM (2): 0.51, 95% CI −0.72 to 1.73; *p* = 0.42 APC + EMD vs. EMD: PDred (2): 1.13, 95% CI −0.05 to 0.30; *p* = 0.16 P (1): −0.10, 95% CI −1.32 to 1.12; *p* = 0.87 SM (1): 0.13, 95% CI −0.05 to 0.31; *p* = 0.15 CAL gain (2): 0.10,95% CI −0.13 to 0.32; *p* = 0.40 P (1): −0.20, 95% CI −1.06 to 0.66; *p* = 0.65 SM (1): 0.12, 95% CI −0.12 to 0.36; *p* = 0.32 BF (1): −0.60%, 95% CI −6.21% to 5.01%; *p* = 0.83	There is very low-quality evidence that the adjunct of APC to OFD or OFD + graft when treating infrabony defects may improve probing pocket depth, clinical attachment level, and radiographic bone defect filling. For GTR or EMD, insufficient evidence of an advantage in using APC was observed.
Li et al., 2019 [29]	12 RCTs	PRF	PRF alone PRF vs. OFD (12) Other comparisons (not meta-analyzed): PRP vs. OFD T-PRF vs. OFD Other comparisons: MF vs. OFD PRF + MF vs. OFD PRF + ATV vs. OFD PRF + RSV vs. OFD	9–12	Yes	287/287	PRF vs. OFD CAL gain (12): 1.29; 95% CI 0.–1.61; *p*< 0.00001 PDred (12): 1.01; 95% CI 0.95– 1.08; *p*< 0.00001 RECinc (8): 0.45; 95% CI 0.31–0.58; *p*< 0.00001 IBDred (8): 1.73; 95% CI 1.38–2.08; *p* < 0.00001 BF (8): 36.47; 95% CI 31.85–41.08; *p*< 0.00001	Adjunctive use of PRF with OFD significantly improves fill defects when compared to OFD alone. However, additional powered studies with much larger sample sizes are needed to obtain a more concrete conclusion. Although the interpretation of the study results was limited, we believe that to a certain extent, our analyses may provide valuable information for physicians who need to decide the best treatment strategy among all possible regimens for patients with intrabony defects.
Baghele et al., 2019 [33]	25 RCTs	PRP PRF	APC alone PRF vs. OFD (13) PRP vs. OFD (3) Other comparisons (not meta-analyzed): T-PRF vs. OFD PRF + HA vs. OFD PRF + MF vs. OFD PRF + ABBM vs. OFD PRF + RSV vs. OFD PRF + ATV vs. OFD PRP + DFDBA vs. OFD PRP + DFDBA vs. PRP (2) PRP + ABBM/P-15 vs. PRP PRF + ABBM vs. PRF PRF + EMD vs. PRF PRF vs. ABG (2) PRF vs. PRP PRF vs. DFDBA MF vs. OFD	6–18	Yes	504/501	APC vs. OFD CAL gain (16): 0.39 (95% CI, 0.35, 0.43, *p* < 0.00001) Pdred (16): 0.68 (95% CI, 0.63, 0.73, *p* < 0.00001) IBDred (13): 1.65 (95% CI, 1.57, 1.73, *p* < 0.00001) RECinc (13): 0.24 (95% CI, 0.22, 0.26, *p* < 0.00001)	Considering all the limitations, we conclude that, use of platelet concentrates (PRF/PRP) as sole grafting agents in periodontal intrabony defects does have an identifiable superiority over not using them during access flap surgeries in terms of only intrabony defect fill. The superiority in terms of clinical parameters (CAL gain, PDred, and RECinc) is negligible. Therefore, use of PRP/PRF can be recommended, with some reservations, as sole grafting material considering its potential for bone fill irrespective of negligible CAL gain and PDred. Considering overall moderate effect sizes in favor of PRP/PRF even for BF, the recommendation should be taken with caution. We did not find any outcome to recommend positively use of PRF/ PRP technologies for treating periodontal intrabony defects if your aim is CAL gain and PDred. There is a need to identify biological cascades and other related factors which are responsible for a wide range of almost negative to highly superior results among analyzed studies. A dedicated large sample size RCT should be carried out to substantiate the findings of this meta-analysis.

ABBM = anorganic bovine bone mineral; ABG = autogenous bone graft; AM = amnion membrane; APC = autologous platelet concentrate; ATV = atorvastatin; BF = bone fill; BG = bioactive glass; BR = bone resorption; CAL gain = clinical attachment level gain; COL = collagen membrane; CI = confidence interval; DFDBA = demineralized freeze-dried bone allograft; EMD = enamel matrix protein derivative; e-PTFE = expanded polytetrafluoroethylene membrane; GTR = guided tissue regeneration; HA = hydroxyapatite; IBDred = intrabony defect depth reduction; MF = metformin; MTB = mandibular taurine bone; NA = not available; NSS = not statistically significant; OFD = open flap debridement; P = parallel groups; p-15 = peptide-15; PAM = polylactic acid membrane; PDred = pocket depth reduction; PP = platelet pellet; PRF = platelet-rich fibrin; PRGF = plasma rich in growth factors; PRP = platelet-rich plasma; RECinc = recession increase; RST = rosuvastatin; SDM = standardized mean difference; SM = split mouth; SS = statistically significant; T-PRF = titanium platelet-rich fibrin; β-TCP = β-tricalcium phosphate. * data provided by the authors.

**Table 5:** Overall effect size for CAL gain.

Comparison	Systematic Reviews	Studies Included	Defects (Test/Control)	Effect Size: SMD (95% CI)	Statistical Significance	*p*-Value
PRP vs. OFD	Panda et al., 2016 [31]	1	18/18 *	0.10 (95% CI: −0.09; 0.29) *	No	0.30 *
PRF vs. OFD	Shah et al., 2014 [25]	5	103/102	0.95 (95% CI: 0.20, 1.71)	Yes	<0.001 *
	Panda et al., 2016 [31]	4	81/80 *	1.48 (95% CI: 1.16, 1.79)	Yes	0.003 *
	Castro et al., 2017 [27]	6	129/129	1.20 (95% CI: 0.5, 1.9)	Yes	<0.001
	Li et al., 2019 [29]	12	287/287	1.29 (95% CI: 0.96–1.61)	Yes	0.00001
PRP + graft vs. graft	Del Fabbro et al., 2011 [23]	6	113/115	0.84 (95% CI: 0.27, 1.42)	Yes	0.004
	Panda et al., 2016 [31]	7	141/140 *	2.00 (95% CI: 1.68, 2.32)	Yes	<0.001 *
	Zhou et al., 2018 [32]	4 **	76/76	0.80 (95% CI: 0.27, 1.32	Yes	NA
	Hou et al., 2016 [24]	8	151/149	1.22 (95% CI: 0.88, 1.57)	Yes	<0.00001
PRP + GTR vs. GTR	Del Fabbro et al., 2011 [23]	4	66/66	0.04 (95% CI: −0.33, 0.41)	No	0.75
	Panda et al., 2016 [31]	4	66/66 *	0.03 (95% CI: −0.32, 0.37)	No	0.74 *
	Hou et al., 2016 [24]	4	66/66	0.08 (95% CI: −0.30, 0.46)	No	0.67
PRF + graft vs. graft	Zhou et al., 2018 * [32]	2	40/40	1.61 (95% CI: 1.10, 2.12)	Yes	NA
APC vs. OFD	Del Fabbro et al., 2018 [22]	12	255/255	1.47 (95% CI 1.11 to 1.82)	Yes	<0.00001
	Baghele et al., 2019 [33]	16	504/501	0.39 (95% CI, 0.35, 0.43)	Yes	<0.00001
APC + graft vs. graft	Del Fabbro et al., 2018 [22]	12	284/284	0.72 (95% CI 0.43 to 1.00)	Yes	<0.00001
APC + GTR vs. GTR	Del Fabbro et al., 2018 [22]	7	124/124	0.42 (95% CI −0.02, 0.86)	No	0.060
APC + EMD vs. EMD	Del Fabbro et al., 2018 [22]	2	38/37	0.10 (95% CI−0.13, 0.32)	No	0.40

PRP = platelet-rich plasma; PRF = platelet-rich fibrin; OFD = open flap debridement; GTR = guided tissue regeneration; NA = not available; SDM = standardized mean difference; CI = confidence interval. * data provided by the authors. ** only DFDBA considered by the authors.

**Table 6:** Quality assessment of the systematic reviews included following the AMSTAR (“A Measurement Tool to Assess Systematic Reviews”) checklist.

Authors and Year	‘A Priori’ Design Provided?	Duplicate Study Selection and Data Extraction?	Comprehensive Literature Search?	Status of Publication (i.e., Grey Literature) as an Inclusion Criterion?	List of Studies (Included and Excluded)?	Characteristics of the Included Studies?	Quality of Included Studies Assessed and Documented?	Scientific Quality Used Appropriately in Formulating Conclusions?	Appropriate Methods Used to Combine the Findings of Studies?	Likelihood of Publication Bias Assessed?	Conflict of Interest Stated?	AMSTAR Score Mean (SD)
Kotsovilis et al., 2010 [21]	1	2	2	2	2	2	2	2	NA	NA	2	17
Del Fabbro et al., 2011 [23]	N	2	2	2	2	1	1	1	1	2	N	14
Del Fabbro et al., 2013 [30]	N	2	2	2	2	1	1	2	N	1	N	13
Shah et al., 2014 [25]	N	2	1	1	N	2	2	1	1	N	N	10
Hou et al., 2016 [24]	N	2	N	1	2	1	1	1	2	2	N	12
Panda et al., 2016 [31]	1	2	2	N	2	1	2	2	1	2	N	15
Castro et al., 2017 [27]	1	2	2	N	2	2	2	1	2	N	N	14
Miron et al., 2017 [28]	1	2	2	N	N	1	N	N	NA	NA	N	6
Najeeb et al., 2017 [26]	1	1	N	N	N	1	2	2	NA	NA	N	7
Del Fabbro et al., 2018 [22]	2	2	2	2	2	2	2	2	2	2	2	22
Saleem et al., 2018 [34]	1	2	2	2	2	1	1	2	2	1	N	16
Zhou et al., 2018 [32]	N	2	N	N	N	1	2	1	2	2	N	10
Baghele et al., 2019 [33]	1	N	2	1	N	1	2	1	1	2	N	11
Li et al., 2019 [29]	N	2	N	1	N	2	2	1	2	N	N	10
AMSTAR score	9	25	19	14	16	19	22	19	16	14	4	12.6 (4.2)

N = criterion not met; NA = not applicable.
